# Antioxidant and Antimicrobial Properties and GC-MS Chemical Compositions of Makwaen Pepper *(Zanthoxylum myriacanthum*) Extracted Using Supercritical Carbon Dioxide

**DOI:** 10.3390/plants12112211

**Published:** 2023-06-03

**Authors:** Sudarut Nadon, Noppol Leksawasdi, Kittisak Jantanasakulwong, Pornchai Rachtanapun, Warintorn Ruksiriwanich, Sarana Rose Sommano, Amin Mousavi Khaneghah, Juan M. Castagnini, Francisco J. Barba, Yuthana Phimolsiripol

**Affiliations:** 1 Faculty of Agro-Industry, Chiang Mai University, Chiang Mai 50100, Thailand; sudarutcat6@gmail.com (S.N.); noppol.l@cmu.ac.th (N.L.); kittisak.jan@cmu.ac.th (K.J.); pornchai.r@cmu.ac.th (P.R.); 2 Cluster of Agro Bio-Circular-Green Industry, Chiang Mai University, Chiang Mai 50100, Thailand; warintorn.ruksiri@cmu.ac.th (W.R.); sarana.s@cmu.ac.th (S.R.S.); 3 Faculty of Pharmacy, Chiang Mai University, Chiang Mai 50200, Thailand; 4 Faculty of Agriculture, Chiang Mai University, Chiang Mai 50200, Thailand; 5 Department of Fruit and Vegetable Product Technology, Prof. Wacław Dąbrowski Institute of Agricultural and Food Biotechnology—State Research Institute, 36 Rakowiecka St., 02-532 Warsaw, Poland; mousavi.amin@gmail.com; 6 Department of Technology of Chemistry, Azerbaijan State Oil and Industry University, 16/21 Azadliq Ave, AZ1010 Baku, Azerbaijan; 7 Research Group in Innovative Technologies for Sustainable Food (ALISOST), Department of Preventive Medicine and Public Health, Food Science, Toxicology and Forensic Medicine, Faculty of Pharmacy, Universitat de València, Avenida Vicent Andrés Estellés s/n, 46100 Burjassot, Spain; juan.castagnini@uv.es (J.M.C.); francisco.barba@uv.es (F.J.B.)

**Keywords:** supercritical fluid extraction, makwaen pepper, *Zanthoxylum myriacanthum*, green extraction, hydro-distillation, antimicrobial property, antioxidant

## Abstract

This research aimed to optimize pressure (10–20 MPa) and temperature (45–60 °C) conditions for supercritical fluid extraction (SFE) of Makwaen pepper (*Zanthoxylum myriacanthum*) extract (ME) in comparison to conventional hydro-distillation extraction. Various quality parameters, including yield, total phenolic compounds, antioxidants, and antimicrobial activities of the extracts, were assessed and optimized using a central composite design. The optimal SFE conditions were found to be 20 MPa at 60 °C, which resulted in the highest yield (19%) and a total phenolic compound content of 31.54 mg GAE/mL extract. IC_50_ values for DPPH and ABTS assays were determined to be 26.06 and 19.90 μg/mL extract, respectively. Overall, the ME obtained through SFE exhibited significantly better physicochemical and antioxidant properties compared to ME obtained through hydro-distillation extraction. Gas chromatography-mass spectrometry (GC-MS) analysis revealed that beta-pinene was the major component in the ME obtained through SFE (23.10%), followed by d-limonene, alpha-pinene, and terpinen-4-ol at concentrations of 16.08, 7.47, and 6.34%, respectively. On the other hand, the hydro-distillation-extracted ME showed stronger antimicrobial properties than the SFE-extracted ME. These findings suggest that both SFE and hydro-distillation have the potential for extracting Makwaen pepper, depending on the intended purpose of use.

## 1. Introduction

**Figure 1:**
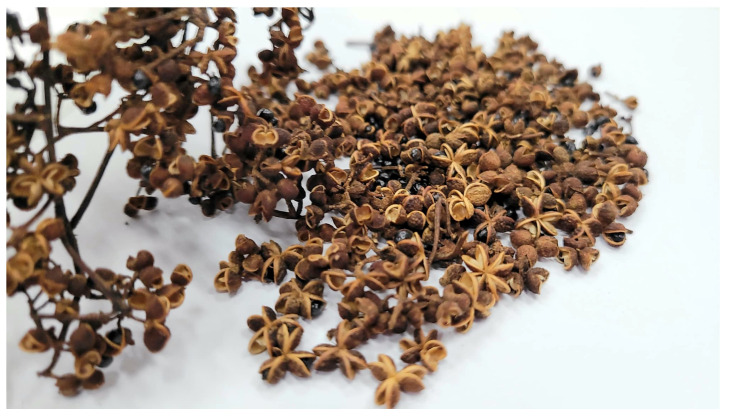
Makwaen pepper.

Makwaen pepper (Figure 1), scientifically known as *Zanthoxylum myriacanthum* (Ma-Kwan in Thai), is a tropical plant primarily found in the northern region of Thailand [1]. Belonging to the Rutaceae family, this plant is widely recognized for its hot and pungent flavor, making it a popular spice in various culinary applications [2]. The genus *Zanthoxylum* comprises more than 250 known species, mainly distributed across tropical and temperate regions of Asia, North America, South America, Africa, and Australia [3,4]. Distinct from other spices, Makwaen pepper possesses a unique and robust scent. Consequently, local communities prefer using its fresh and dried fruits as a traditional spice to add spiciness and enhance the flavor of their meals. This preference is attributed to the pleasant aroma of Makwaen pepper, which can stimulate appetite. It is a staple ingredient in many traditional Thai dishes, including spicy pork salad, vegetable soup, and chili paste [2].

Makwaen pepper, a member of the Rutaceae family, is rich in essential oils found in its leaves and fruits [5]. The main constituents of the oil derived from Makwaen pepper include 67.06% limonene, 6.49% α-pinene, 3.87% β-myrcene, and 2.96% linalool. Additionally, the essential oil can be found in the seed coats, seeds, and whole fruits of Makwaen pepper. It is worth noting that the content of limonene, a major chemical component, varies across different parts of the plant [6]. Traditionally, Makwaen pepper has been highly valued as both a spice and herbal remedy. Local wisdom has recognized its effectiveness in treating a wide range of conditions, including abdominal pain, arthritis, asthma, cough, cold, fever, diarrhea, malaria, toothache, and ascarid infections, as well as muscle cramps and spasms [7]. The essential oil derived from Makwaen pepper plays a vital role in traditional medicine, serving as an active ingredient in analgesic, anticonvulsant, and anthelmintic herbal drugs. Moreover, this essential oil exhibits various beneficial properties, such as anti-inflammatory, antinociceptive, antioxidant, antiparasitic, antitumor, and antimicrobial activities [8].

Several publications have highlighted the intriguing process of extracting essential oil from Makwaen pepper [2,6,9]. Various methods have been employed, including soaking, maceration, pressurized hot water extraction, Soxhlet solvent extraction, and hydro-distillation, to recover bioactive compounds from Makwaen pepper [10]. However, it is important to note that many of these extraction methods involve the use of organic solvents such as hexane, toluene, chloroform, methanol, and acetone. Unfortunately, the use of these solvents can result in the generation of harmful and toxic byproducts and waste materials. Proper treatment and disposal of these byproducts and wastes are necessary to mitigate environmental impacts and ensure the safety of the extraction process [11]. Therefore, there is a need for alternative extraction methods that minimize the use of organic solvents and offer more sustainable and environmentally friendly approaches for essential oil extraction from Makwaen pepper.

Recently, supercritical fluid extraction (SFE) using CO_2_ has emerged as a promising and innovative technology for extracting bioactive compounds, including essential oils, from various natural sources. This method offers several advantages over traditional extraction techniques, making it a preferred choice in recent years [12,13]. Supercritical fluids, such as CO_2_, possess unique physicochemical properties that lie between those of gases and liquids [14]. Specifically, the critical region of CO_2_ occurs at 31.1 °C and 73 atm, where a dynamic equilibrium is established, and there is no clear distinction between the liquid and gas phases [15]. When CO_2_ is in its supercritical state, it surrounds the solid molecules or particles of the target material, leading to interactions that decrease the enthalpy energy and facilitate dissolution. Furthermore, supercritical fluids exhibit viscosity and diffusion coefficients similar to gases, allowing them to efficiently penetrate and diffuse through the solid structure of materials. As a result, supercritical fluids are highly effective as extraction solvents, offering advantages such as rapid mass transfer rate and greater solubility compared to traditional liquid solvents [16].

Certainly, ensuring the safety and quality of essential oils from Makwaen pepper is crucial for their diverse applications, including aromatherapy, cosmetics, and medicinal and food products. It is essential to obtain high-quality essential oils without impurities while preserving their antioxidant and antimicrobial activities. Therefore, the objective of this research was to identify an appropriate extraction method that yields desirable essential oils from Makwaen pepper.

## 2. Results and Discussion

### 2.1. Effect of SFE Parameters on Extract Yield 

Table 1 presents the percentage yields of the ME obtained through SFE, ranging from 9.24 to 20.49%. The regression equation analysis demonstrated that the extract yield was significantly influenced by the extraction temperature and pressure, with a significance level of *p* < 0.05. As depicted in Figure S1, the relationship between the extraction yield, temperature, and pressure exhibited a linear pattern with an *R*^2^ value of 0.875. Increasing the pressure (ranging from 8 to 22 MPa) positively affected the extract yield, indicating a direct correlation between pressure and the percentage yields of the ME. Conversely, an increase in temperature displayed a negative correlation with the percent yields of ME. This observation aligns with the findings of Majdoub et al. [3], who reported a significant enhancement in extraction yield with increased pressure at a constant temperature. The higher yield can be attributed to the increased solubility of the compounds under high-pressure conditions. The reduction in the surface between CO_2_ molecules at higher pressure enhances their density and facilitates better contact between the extractable compounds and the extraction solvent [17].

Based on the response surface contour plots in three dimensions, it was observed that increasing the temperature at both low and high pressures slightly enhanced the yield of ME. This might be due to the influence of the vapor pressure of supercritical CO_2_, which aids in facilitating diffusion. However, it was determined that temperature did not play a dominant role in this experiment for achieving a higher extract yield [18].

### 2.2. Effects of Process Variables on Total Phenolic Compounds

The total phenolic compounds (TPC) of ME obtained through SFE are presented in Table 1. The relationships between TPC and extraction temperature and pressure are depicted in Figure S1, demonstrating a linear model with an impressive *R*^2^ value of 0.997. Regression analysis was performed on the experimental data, and the coefficients of the model were evaluated for significance. The results revealed that the extraction temperature had a highly significant effect (*p* < 0.05) on the extraction of phenolic compounds. The TPC of ME obtained using SFE ranged from 14.02 to 31.04 mg GAE/mL, as shown in Table 1. This increase in TPC can be attributed to the enhanced solvating power of CO_2_ resulting from high pressure and temperature.

### 2.3. Antioxidant Properties of ME

Antioxidation is a complex process that typically involves various mechanisms. Consequently, the assessment of antioxidant activity in extracts from natural sources generally employs multiple evaluation methods. The analytical results for antioxidant activity utilizing DPPH, ABTS, and FRAP assays for ME obtained through SFE are presented in Table 1. The result of the IC_50_, calculated using the DPPH assay, indicates that the IC_50_ values for the ME ranged from 31.62 to 97.80 µg/mL extract, denoting higher antioxidant activity as the values decreased. Regression analysis highlights that the primary extraction parameter influencing the DPPH radical scavenging activity of ME was the extraction temperature (*p* < 0.05). Additionally, the quadratic terms of the extraction process variables significantly impacted the antioxidant activities of the extract. The regression equation for DPPH exhibiting a strong fit with an *R*^2^ value of 0.990 is presented in Table S1, indicating its reliability.

It can be observed that there is a correlation between the total phenolic compounds present in extracts and their antioxidant activities. Various researchers have documented consistent linear relationships between phenolic compounds and antioxidant properties. The radical scavenging ability of different extracts of *Z. myriacanthum* is likely attributed to the presence of polyphenols, flavonoids, and other phenolic compounds. Phenolics contribute significantly to the antioxidant activity found in plants [19]. Additionally, the DPPH radical scavenging model is widely employed to assess the capability of natural compounds to eliminate free radicals. In this assay, antioxidants effectively reduced the stable DPPH radical, resulting in the formation of a yellow-colored diphenyl picryl hydrazine. The ability of antioxidants to scavenge DPPH radicals is believed to stem from their capacity to donate hydrogen atoms [20].

The ABTS assay was used to calculate the IC_50_ of the ME, which ranged from 25.05 to 73.55 µg/mL extract. A sample demonstrating a potent inhibitory effect on free radical oxidation slows down the rate of color change in the solution, turning it bluish-green. Regression analysis of the ABTS assay revealed that the key extraction parameters for the ME were primarily influenced by the extraction temperature, which had a highly significant effect. The antioxidant activities of the ME were affected by the quadratic terms of the extraction process variables, resulting in an impressive *R*^2^ value of 0.998, presented in Table S1. This value, close to 1, indicates a strong correlation between the experimental and predicted values.

The FRAP assay is commonly employed to assess the ability of antioxidants to donate electrons. The ME exhibited an interesting antioxidant effect in terms of its reducing power, ranging from 140.78 to 190.48 mg FeSO_4_/mL under different temperatures and pressures. However, regression analysis indicated that the main extraction parameters for the FRAP assay from the ME were not statistically significant (*p* ≥ 0.05). The antioxidant activities of the ME were affected by the two-factor interaction (2FI) terms of the extraction process variables, resulting in an *R*^2^ value of 0.366, presented in Table S1. The FRAP assay is particularly suitable for measuring the antioxidant activity of water-mediated compounds or those containing a significant water-based structure [21]. *Zanthoxylum* oil consists of alkaloids that have the potential to interact with Fe^3+^ ions and interfere with the reduction reaction. Therefore, there was no significant difference in the ferric reducing ability of ME (*p* ≥ 0.05).

Table S2 displays the results of Pearson correlation analysis, revealing a correlation between TPC and IC_50_ of DPPH and ABTS. The correlation observed is inverse (−), with Pearson correlation coefficients of (−0.654) and (−0.783), respectively. The decreasing IC_50_ values for both DPPH and ABTS indicate a higher antioxidant capacity. Moreover, the relationship between IC_50_ values of DPPH and ABTS demonstrates a positive correlation (+), as indicated by a Pearson correlation coefficient of 0.841. This suggests that when the IC_50_ value of DPPH decreases, the IC_50_ value of ABTS also decreases. Conversely, the correlation between TPC and FRAP was in the opposite direction (−), with a Pearson correlation coefficient of −0.279. This indicates that an increase in phenolic content leads to a decrease in FRAP values.

### 2.4. Antibacterial Activity of ME

Table 2 presents the results of the agar disc diffusion method used to evaluate the antibacterial activity of ME obtained from SFE against various bacteria. The tested bacteria include gram-positive strains such as *S. aureus and B. subtilis*, as well as gram-negative strains such as *P. aeruginosa* and *E. coli*. A concentration of 1 mg/L ME was used in the experiments. The findings indicate that the ME obtained through SFE exhibited greater efficacy against gram-positive bacteria compared to gram-negative bacteria. Furthermore, an observation from the trend of extraction parameters revealed that the antibacterial effect of the ME increased with higher temperatures.

The agar disc diffusion method was utilized to conduct preliminary tests on the inhibition (clear zone) and determine the antimicrobial activity of ME extracted by SFE against both gram-positive and gram-negative bacteria. The results of this investigation demonstrated that the ME obtained through SFE effectively inhibited the growth of *S. aureus*, with the maximum zone of inhibition ranging from 11.33 ± 0.05 mm to 21.67 ± 0.27 mm. It is widely known that hydrophobic compounds, including essential oils, have a greater impact on gram-positive bacteria [22]. Gram-positive bacteria possess a thick peptidoglycan layer, which readily interacts with other hydrophobic molecules such as teichoic acid and proteins. This hydrophobic layer around their cells may facilitate the entry of hydrophobic molecules. On the other hand, gram-negative bacteria have an outer membrane consisting of proteins and lipopolysaccharides, making them more resistant to hydrophobic compounds present in essential oils [23]. 

Therefore, the MIC of the ME was determined to further assess its effectiveness. The study employed the highest initial value of 80% (*v*/*v*) for testing. Table 2 displays the MIC values for susceptible bacteria, including *E. coli*, *S. aureus, P. aeruginosa*, and *B. subtilis*. Additionally, the MIC test results were used to determine the MBC of the ME at each concentration, as shown in Table 3. Interestingly, there were no significant differences observed between the MIC values of gram-positive and gram-negative bacteria. Van de Vel et al. [24] reported that certain essential oil molecules exhibit higher activity against gram-positive bacteria, while others are effective against gram-negative bacteria. In most studies investigating the antimicrobial properties of essential oils, *E. coli* and *S. aureus* have been commonly employed as model microorganisms representing gram-negative and gram-positive bacteria, respectively. The potential mechanisms by which the ME inhibits bacterial growth include (1) disruption of the bacterial outer membrane or phospholipid bilayer, (2) modification of fatty acid composition, (3) induction of membrane fluidity leading to potassium ion and proton leakage, (4) hindrance of glucose uptake, and (5) inhibition of enzyme activity or cell lysis [23,25].

### 2.5. Optimization of the Extraction Process

**Figure 2:**
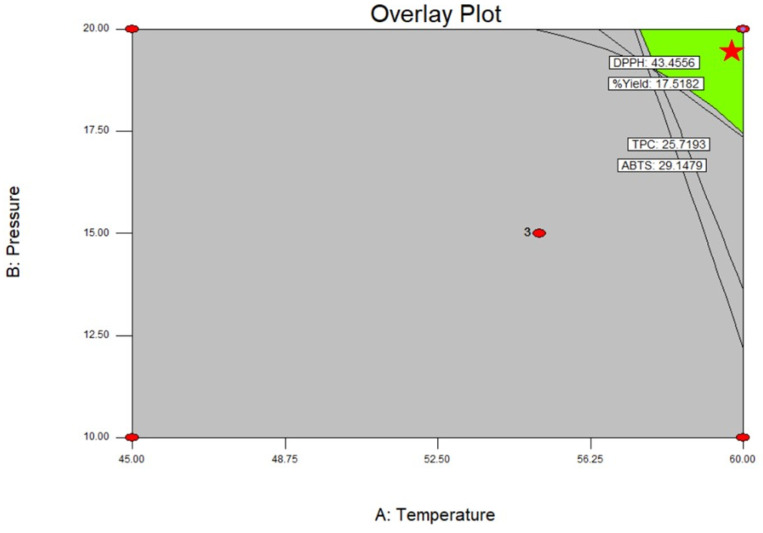
Overlay plot of the response surface regression model between the temperature and pressure.

To optimize the conditions of SFE for achieving desirable extract yields, total phenolic compounds, and antioxidant activities in the ME, the central composite design (CCD) was employed. Through this design, it was determined that an SFE temperature of 60 °C and pressure of 20 MPa, as depicted in Figure 2, could yield optimal results, including an extract yield of 18.99%, total phenolic compounds of 27.48 mg GAE/mL, and DPPH and ABTS antioxidant activities of 28.64 µg/mL and 37.46 µg/mL, respectively. The predicted results closely matched the experimental results obtained using the optimum extraction conditions, thereby confirming the validity of the CCD model and demonstrating a strong correlation. Additionally, the desirability function approach was employed to predict a set of optimal conditions for all four response variables.

### 2.6. GC-MS Chemical Compositions

**Figure 3:**
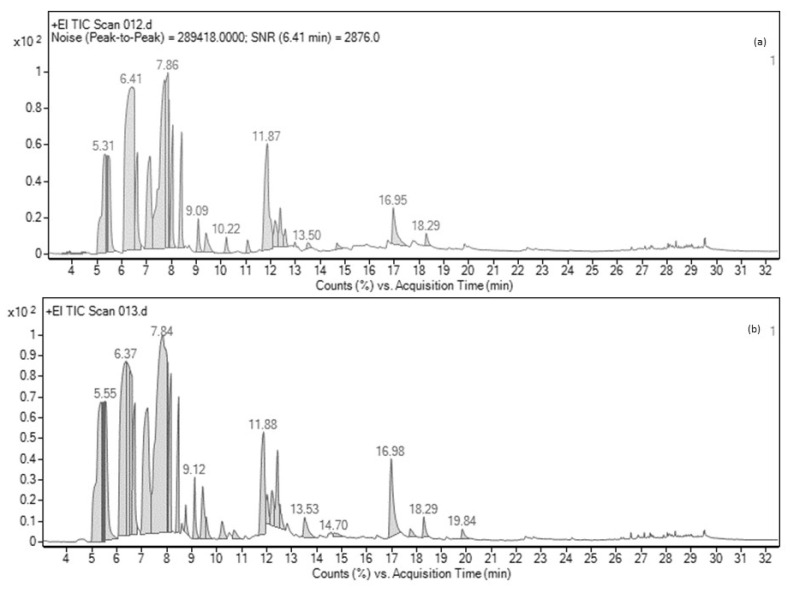
Gas chromatography fingerprinting of (**a**) supercritical fluid extracted ME and (**b**) hydro-distilled ME.

The composition of essential oils obtained through supercritical fluid extraction (SFE) and hydro-distillation was analyzed using GC-MS. The results, presented in Table 4 and Figure 3, revealed that the essential oil extracted via SFE contained a mixture of 23 different chemical components. The primary constituent was beta-pinene, comprising 23.10% of the oil, followed by d-limonene at 16.08%. Additionally, alpha-pinene and terpinen-4-ol accounted for 7.47 and 6.34% of the composition, respectively. On the other hand, the essential oil obtained through hydro-distillation comprised about 25 components. The major component, in this case, was d-limonene, making up 22.94% of the oil. It was followed by beta-pinene, alpha-pinene, alpha-phellandrene, and sabinene at percentages of 11.20%, 9.37%, 8.61%, and 3.87%, respectively.

The results indicated that the variations in the composition of ME obtained from the same plant could stem from different extraction methods. Beta-pinene is the main component of ME extracted by SFE, whose extraction effectiveness can be influenced by various factors, including extraction parameters temperature, pressure, plant species, and the desired target compounds. The solvating power of supercritical CO_2_ can be adjusted by modifying the temperature and pressure, which allows for the selective extraction of specific compounds [26]. For SFE, the non-polar nature of CO_2_ is considered suitable for certain types of chemical components with higher affinity. These include non-polar compounds such as terpenoids and essential oils, which are more readily soluble in supercritical CO_2_. On the other hand, polar compounds such as certain flavonoids and alkaloids may have lower solubility in CO_2_ alone and may require additional methods or modifiers to enhance their extraction efficiency [27]. In fact, the polar nature of hydro-distillation also enables the extraction of a broad range of polar and non-polar compounds from *Z. myriacanthum,* such as limonene, which is also another major component of ME [26,28]. Zhang et al. [28] also compared the SFE and hydro-distillation methods for *Z. bungeanum* extraction and reported higher extraction efficiency of monoterpene-ester components from the former method. The relatively lower temperature applied in SFE compared with hydro-distillation might help preserve the integrity of thermally sensitive compounds and thus minimize chemical composition changes.

According to Wongkattiya et al. [2], the active components responsible for inhibiting bacteria, such as *S. aureus, E. coli, P. aeruginosa*, and *B. subtilis*, in the hydro-distilled ME were sabinene, terpinen-4-ol, d-limonene, and alpha-pinene. Among these, sabinene was identified as the most significant compound with the ability to inhibit a broad range of bacteria. Despite its relatively low concentration in the essential oil extract, the ME exhibited inhibitory effects on the growth of *B. subtilis* and *E. coli*. D-limonene and alpha-pinene demonstrated stronger inhibitory effects on gram-positive microorganisms (*B. subtilis* and *S. aureus*) compared to gram-negative bacteria, which is consistent with the findings of Tangjitjaroenkun et al. [3]. Their research focused on the properties of *Z. myriacanthum* fruit extracts obtained through hydro-distillation and pure major compounds (sabinene, limonene, and terpinene-4-ol). The extracts were evaluated using paper disk diffusion against bacteria *B. subtilis, S. aureus, E. coli*, and *P. aeruginosa.* The study revealed that sabinene exhibited potent antibacterial activity against *B. subtilis, S. aureus*, and *E. coli,* but its effectiveness against *P. aeruginosa* was weaker. Okagu et al. [29] also extracted essential oils from *Z. myriacanthum* using hydro-distillation and investigated the compositions by GC-MS. They indicated that the predominant components in the essential oil were limonene (62.51%) and sabinene (13.83%). In addition, the research conducted by Supabphol and Tangjitjaroenkun [30], as well as Sriwichai et al. [31], reported that limonene and sabinene were major components of the essential oil extracted from *Z. myriacanthum*.

### 2.7. Comparison of Supercritical Fluid Extraction with the Conventional ME

#### 2.7.1. Antioxidant Activity

**Figure 4:**
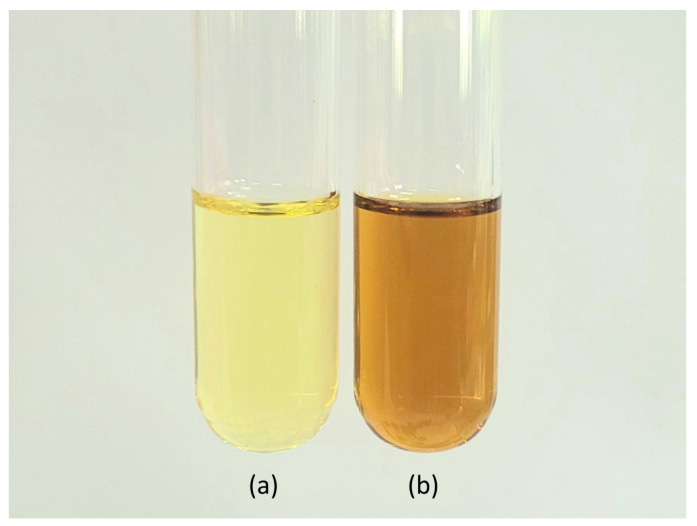
Appearance of ME extracted by (**a**) hydro-distillation and (**b**) supercritical fluid extraction.

The optimal conditions for extracting the ME through SFE were determined by considering both physicochemical characteristics and antioxidant activity. A comparison between ME obtained through SFE and hydro-distillation extraction is presented in Figure 4 and Table 5. The results showed that the ME obtained through SFE exhibited higher percentage yield, phenolic compound content, and antioxidant activity compared to the hydro-distillation extraction method. Moreover, the physical and chemical properties of the ME obtained through SFE were superior to those obtained through hydro-distillation. Notably, SFE demonstrated a significant advantage in terms of extraction time, saving more than 3 h per extraction when compared to conventional extraction methods, which typically require around 4 h per extraction.

The comparison of antioxidant activities of ME extract from different methods is shown in Table 5. Implementation of SFE and hydro-distillation resulted in significantly different (*p* < 0.05) antioxidant activities for total phenols, IC_50_ DPPH and IC_50_ ABTS. The total phenols of ME extracted by hydro-distillation were significantly higher (*p* < 0.05) than that of SFE. This could be related to thymol content as measured by GC-MS, as presented in Table 5, with a relatively higher peak area from the hydro-distillation method.

For IC_50_ of DPPH and ABTS, the ME extract obtained from SFE had lower IC_50_ values than those from hydro-distillation, which could be interpreted as having higher antioxidant activity. The presence of high beta-pinene content in ME extracted by SFE (Table 4) might contribute to the greater antioxidant activities. Beta-pinene is a biologically active compound and other important monoterpene constituents of essential oils [32,33]. In addition, beta-myrcene and terpinen-4-ol are also key compositions with strong antioxidant activity in ME extract from SFE. It was reported that most monoterpenes had relatively high free radical scavenging activity in ME [34].

#### 2.7.2. Antimicrobial Activity

Table 6 presents a comparison of the antimicrobial activity of ME obtained through different extraction methods. Both hydro-distillation and SFE yielded ME with antimicrobial activity against gram-positive and gram-negative bacteria. The ME demonstrated broad-spectrum antibacterial activity against *S. aureus*, *E. coli*, *B. subtilis*, and *P. aeruginosa.* The antimicrobial efficacy of ME is influenced by various factors, including its chemical composition, extraction methodology, and the nature of the target gram-positive (*B. subtilis,* and *S. aureus*) or gram-negative bacteria (*P. aeruginosa*, and *E. coli*). Comparatively, ME obtained through hydro-distillation showed greater effectiveness than SFE due to the presence of sabinene, a potent antimicrobial compound. The ME contains terpene compounds such as beta-caryophyllene, p-cymene, alpha-pinene, beta-pinene, limonene, sabinene, gamma-terpinene, alpha-terpinene, and beta-myrcene, which contribute to its antimicrobial activity [23,30].

## 3. Materials and Methods

### 3.1. Plant Materials, Cultures, and Chemicals

Makwaen fruits were purchased from Nanthachai Solutions Ltd., Part., located in the Pha Sing sub-district, Mueang Nan district, Nan province, Thailand. To prepare the fruits for extraction, those without stalks were subjected to a drying process at 60 °C using a hot air oven (ED56, Binder GmbH, Tuttlingen, Germany). Dried fruits were removed from the oven once a constant weight was reached, corresponding to a dry weight reduction of 10%. Afterward, the dried fruits were carefully stored and utilized as the substrate for essential oil extraction within a period of three months.

The 2,2-Diphenyl-1-picrylhydrazyl (DPPH), 2,2-azino-bis (3-ethylbenzothiazoline-6-sulfonicacid (ABTS), 2,4,6-tris(2-pyridyl)-s-triazine (TPTZ), and gallic acid (GA), were purchased from Sigma–Aldrich (Singapore). Hydrochloric acid (HCl) was sourced from RCI Labscan (Bangkok, Thailand). Ferrous sulfate (FeSO_4_) was purchased from Merck KGaA (Darmstadt, Germany). Sodium Carbonate (Na_2_CO_3_) and the Folin–Ciocalteu reagent were bought from Loba Chemie (Mumbai, India). Nutrient broth (NB), Mueller–Hinton broth (MHB), and Mueller–Hinton agar (MHA) were purchased from Himedia (Mumbai, India). Plate count agar (PCA) and peptone water were obtained from Difco (Cockeysville, MD, USA). All other reagents used in this study were of analytical grade.

Four species of bacteria were utilized in this study: *Staphylococcus aureus* (American Type Culture Collection, ATCC 25923), *Escherichia coli* (ATCC 25922), *Bacillus subtilis* (Department of Medical Sciences Thailand, DMST 15896), and *Pseudomonas aeruginosa* (Thailand Institute of Scientific and Technological Research, TISTR 781). These bacterial strains were obtained from the Thailand Institute of Scientific and Technological Research (Pathum Thani, Thailand). Prior to use, all stock cultures of the microorganisms were maintained in glycerol solution (30%, *w*/*w*) and stored at a temperature of −20 °C until use.

### 3.2. Supercritical Fluid Extraction

The extraction of dried Makwaen pepper was conducted using an SFE (Guangzhou Heavensent Industrial Co., Ltd., Guangzhou, China), following the method described by Lopresto et al. [35] with slight modifications. To optimize the extraction conditions, including pressure and temperature, a central composite design (CCD) was employed. For each extraction, 1400 g of dried Makwaen pepper was placed into a 5 L extraction vessel. The liquid CO_2_ stored in the tank was cooled to a temperature range between 2 and 6 °C using a refrigeration system. It was then pressurized to the desired level using a high-pressure diaphragm pump and passed through a heat exchanger to achieve the supercritical phase. Throughout the extraction process, the average flow rate of CO_2_ was maintained at 140 L/h. After extraction, the mixture of the extracted sample and CO_2_ was separated in a separator, with the CO_2_ being recycled back into the storage tank. The yield of Makwaen extract (ME) was determined using Equation (1).

(1) $$Extraction\;yield\left(\%\right)=\frac{Dried\;of\;Makwaen\;pepper\left(g\right)-Makwaen\;pepper\;extracted(g)}{Dried\;of\;Makwaen\;pepper\left(g\right)}\times 100$$

### 3.3. Hydro-Distillation Extraction

The hydro-distillation extraction of Makwaen pepper was carried out following the method outlined by Sriwichai et al. [36]. In this process, 100 g of dried Makwaen pepper was soaked in 600 mL of distilled water. The extraction setup was heated using a heating mantle (MS-E106, Mtops, Kyunggi-do, Korea) and gradually increased to the maximum heating capacity of the mantle, reaching a temperature of 100 °C. The heating was maintained at this temperature for 4 h, allowing the mixture to boil. The yield of the ME was calculated as the percentage obtained from the dry plant material. To remove any residual moisture, the ME was dried using anhydrous sodium sulfate and stored at 4 °C until further analysis. The experimental design for the extraction process is depicted in Figure S2.

### 3.4. Total Phenolic Content (TPC)

The TPC values were determined using the Folin–Ciocalteu colorimetric assay following the method described by Değirmenci and Erkurt [37]. Briefly, 500 µL of the ME (0.0625 mg/mL) was mixed with 2.5 mL of 10% Folin–Ciocalateu reagent and 2 mL of a 7.5% (*w*/*v*) sodium carbonate solution. The mixture was allowed to react for a specified time. The absorbance of the resulting solution was measured at a wavelength of 760 nm using a 96-well microplate reader (Synergy H1M, Bio-Tek, Winooski, VT, USA). The TPC was expressed as mg of gallic acid equivalent per gram of extract (mg GAE/g).

### 3.5. Antioxidant Measurements of ME

#### 3.5.1. DPPH Radical Scavenging Activity

The method for determining the radical scavenging activity of the ME using 2,2-diphenyl-1 picrylhydrazyl (DPPH) was adapted from Chaiwong et al. [38]. In summary, 2 mL of 0.5 mmol/L DPPH dissolved in methanol was combined with 100 µL of varying concentrations of ME (0.0312, 0.0625, 0.125, 0.25, 0.5, and 1 mg/mL). After 30 min of incubation in the darkness, the absorption at 517 nm was measured using a 96-well microplate reader (Synergy H1M, Bio-Tek, Winooski, VT, USA). DPPH is a stable purple free radical, and when antioxidant compounds scavenge free radicals, the color changes to a pale yellow, which can be detected by the microplate reader. The percentage of DPPH radical scavenging activity was calculated using Equation (2) prior to plotting the IC_50_ against the corresponding concentrations.

(2) $$\%\;DPPH\;radical\;inhibition=\frac{\left(A_{517\;con}-A_{517\;sam}\right)}{A_{517\;con}}\times 100$$

where *A*_517 *con*_ is the absorbance of the control reaction (containing all reagents except the test compound), and *A*_517 *sam*_ is the absorbance of the tested sample. Scatter diagrams were plotted, and linear regression was estimated. The IC_50_ value, which corresponds to the concentration causing 50% inhibition of DPPH, was determined through calculations.

#### 3.5.2. ABTS Radical Scavenging Activity

The 2,2-Azino-bis-(3-ethylbenzothiazoline-6-sulfonic acid) (ABTS) radical scavenging activity of the ME was determined following the method of Chaiwong et al. [38] with some modifications. To generate the ABTS radical, an aqueous solution (7 mmol/L) was mixed with K_2_S_2_O_8_ (2.45 mmol/L final concentration) and allowed to react in darkness for 16 h. Subsequently, the absorbance of the reacted solution at 734 nm was adjusted to 0.70 ± 0.02 using ethanol. A suitable dilution (0.2 mL) of the ME extract was added to 2.0 mL of ABTS radical solution. The ABTS assay quantitatively determines the antioxidant activity based on the reaction of the ABTS free radical, which undergoes a color change from deep blue to colorless in the presence of antioxidants. After 15 min of reaction time, the absorbance was measured at 734 nm using a 96-well microplate reader (Synergy H1M, Bio-Tek, Winooski, VT, USA). The percentage of ABTS radical scavenging activity was calculated according to Equation (3), and the IC_50_ values were plotted against the respective concentrations.

(3) $$\%\;ABTS\;radical\;inhibition=\frac{\left(A_{734\;con}-A_{734\;sam}\right)}{A_{734\;con}}\times 100$$

where *A*_734 *con*_ is the absorbance of the control reaction (containing all reagents except the test compound), and *A*_734 *sam*_ is the absorbance of the tested sample. Scatter diagrams were plotted, and linear regression was estimated. IC_50_ was calculated as the concentration that caused 50% inhibition of ABTS.

#### 3.5.3. Ferric Reducing Antioxidant Power

The ferric reducing antioxidant power (FRAP) assay was conducted according to the method outlined by Chaiwong et al. [38] and Toazza et al. [39]. In this assay, the reaction involved a solution of FeCl_3_.6H_2_O in distilled water (with a final concentration of Fe^3+^ at 20 mM), 4,6-tripyridyl-s-triazine TPTZ in 40 mM HCl (with a final concentration of TPTZ at 10 mM), and 0.3 M acetate buffer solution at pH 3.6. The FRAP reagent was prepared by combining an acetic acid buffer, TPTZ solution, and FRAP test solution in a volume ratio of 10:1:1. The reaction mixture was then measured at 593 nm using a 96-well microplate reader (Synergy H1M, Bio-Tek, Winooski, VT, USA). The FRAP value of the sample was determined using ferrous sulfate (FeSO_4_) as the standard. The results were expressed as the FeSO_4_ equivalent antioxidant capacity in the µmol Fe^2+^/g sample.

### 3.6. Antimicrobial Activities

#### 3.6.1. Agar Well Radical Diffusion Assay

The antimicrobial activity was evaluated using the agar well diffusion method, following a modified approach based on Clain et al. [40]. To begin, a bacterial cell suspension was prepared from a 24 h culture. The inoculation density was adjusted to 1 × 10^6^ colony forming units per mL (cfu/mL) using McFarland No. 0.5 as a standard. The bacterial culture was spread evenly on sterile nutrient agar plates using a swab. Subsequently, filter paper discs (6 mm in diameter) were dipped into the ME and positioned onto the agar plates that were previously swabbed. As a positive control, antimicrobial susceptibility discs of Tetracycline (30 mg/mL) (Thermo Fisher Scientific, Waltham, MA, USA) were employed. The plates were incubated in an upright position at 37 °C for 18–24 h. The experiment was carried out in triplicate, and the inhibition zone, expressed in millimeters, was recorded as a measure of antimicrobial activity.

#### 3.6.2. Determination of Minimum Inhibitory Concentration and Minimum Bactericidal Concentration

The determination of the minimum inhibitory concentration (MIC) and minimum bactericidal concentration (MBC) was carried out following a modified approach based on the methods described by Phimolsiripol et al. [41] and Skenderidis et al. [42]. The MIC and MBC tests of the ME were performed using the broth dilution method in test tubes.

The MIC represents the concentration of the ME required to inhibit the growth of the tested microorganism. In this study, the ME was initially prepared at a concentration of 80 mg/mL and then further diluted with Tween 20 to generate two-fold serial dilutions, resulting in concentrations of were done to obtain solutions at 40, 20, 10, 5, 2.5, 1.25, 0.625, 0.312, and 0.156% (*v*/*v*). The initial microbial samples were adjusted to turbidity matching a 0.5 McFarland standard, resulting in a microbial density of ~1 × 10^8^ CFU/mL. Each sample solution (250 µL) was mixed with 250 µL of sterile Mueller–Hinton broth (MHB). The resulting solution was then inoculated with 250 µL of microbial suspension and incubated at 37 °C for 24 h. Changes in turbidity were measured at 600 nm and compared with the control to assess the inhibitory effect of the ME.

The MBC represents the lowest concentration of the ME required to deactivate the tested microorganisms. Following the determination of the MIC, aliquots were taken from all tubes that did not show visible bacterial growth. These aliquots were streaked onto Mueller–Hinton agar plates and then incubated for 24 h at 37 °C. The presence or absence of bacteria was observed on both pre- and post-incubated plates. An MBC endpoint was reached when the lowest concentration of the antimicrobial agent resulted in a 99.9% reduction in the bacterial population.

### 3.7. Gas Chromatographic/Mass Spectrometry (GC/MS) Analysis of Essential Oil Compositions

The optimal extraction of the essential oil using SFE was chosen based on the optimization of yield, antioxidation properties, and antimicrobial activities. A comparison was made with essential extraction using hydro-distillation. The resulting essential oil was then analyzed using GC/MS following a modified method described by Wongkattiya et al. [2] and Charoensup et al. [43]. GC fingerprinting was carried out using Agilent Technology apparatus (Agilent 7890B, USA) equipped with a Hewlett Packard mass selective detector (Agilent 5977B GC/MSD, Santa Clara, CA, USA) and an HP-5MS column (30 m × 0.25 mm id., × 0.25 µm film thickness); (HP-5MS, Santa Clara, CA, USA). The temperature program for the oven was set as follows: 70 °C (0–3 min); 70–188 °C (3 °C/min); 188–280 °C (20 °C/min); 280 °C (3 min). Helium was used as the carrier gas with a flow rate of 1 mL/min, and the injection volume was 1 µL (10 µL of essential oil diluted with 490 µL of dichloromethane). A transfer line connected the MS to the GC, maintaining a temperature of 150 °C, while the ion source temperature was set to 230 °C. Electron ionization mode at 70 eV was used for MS, and the mass range scanned was between 30 and 300 amu. The chemical constituents of ME were identified by comparing their mass spectra and retention indices with the Wiley Mass Spectral Library. The compound identification was further confirmed using the Wiley 275 and NIST98 libraries (John Wiley & Sons, New York, NY, USA).

### 3.8. Statistical Analysis

Design-Expert version 6.0.2 (Design 6.0.2, Stat-Ease, Inc., Minneapolis, MN, USA) was utilized to apply response surface methodology and optimize the experimental data. Regression analysis and response surface plots were employed to determine the optimal extraction conditions by examining the relationship between independent and dependent variables. The data were subjected to one-way analysis of variance (ANOVA), and mean separation was conducted using Duncan’s multiple range tests with a significance level of *p* < 0.05. Significant differences were indicated by using the same letters in a row. Statistical analyses were performed using SPSS 17.0 (SPSS, Inc., IBM Corp., Chicago, IL, USA), and *t*-tests (*p* < 0.05) were used to describe significant differences.

## 4. Conclusions

The optimal extraction of Makwaen pepper using SFE was found to be enhanced with increasing pressure. The ideal SFE conditions were determined to be 60 °C and 20 MPa. A comparison was made between the physical properties and antioxidant activity of the SFE-extracted sample and the hydro-distilled sample. Under the optimum extraction conditions, the SFE method yielded the highest extraction yield (19.19%) and total phenolic compound content (31.54 mg GAE/mL). Additionally, the antioxidant activities measured by IC_50_ values of DPPH and ABTS were significantly higher in the SFE-extracted sample compared to the hydro-distilled sample. SFE proved to be a suitable process for obtaining a high-yield medium enriched with potent antioxidant activities. Furthermore, the extraction time was reduced by 3 h. The resulting medium obtained through the optimal SFE condition demonstrated the ability to inhibit the growth of *S. aureus*, *E. coli*, *B. subtilis,* and *P. aeruginosa.* Therefore, further investigation is warranted to fully explore the properties and potential applications of this medium. In addition, future work is required to investigate the maximum doses used in antimicrobial tests in terms of cytotoxicity and hemolytic activities.

## Figures and Tables

**Table 1:** Physicochemical properties of ME.

Treatment	SFE Conditions		Properties				
	Temperature (C°)	Pressure (MPa)	Extract Yield (%)	Total Phenols (mg GAE/mL)	IC_50_ DPPH (µg/mL Extract)	IC_50_ ABTS (µg/mL)	FRAP ^ns^ (mgFeSO_4_/mL)
1 (−1, −1)	45	10	9.33	14.02 ± 0.13	97.80 ± 1.56	70.82 ± 1.34	157.7 ± 2.37
2 (+1, −1)	60	10	9.33	26.7 ± 1.69	49.87 ± 1.37	30.59 ± 0.84	190.48 ± 1.72
3 (−1, +1)	45	20	11.09	15.73 ± 0.13	48.12 ± 0.24	42.56 ± 0.34	173.86 ± 1.08
4 (+1, +1)	60	20	17.43	31.04 ±1.82	40.43 ± 2.72	28.46 ± 0.26	165.17 ± 0.86
5 (−α, 0)	40	15	7.92	14.92 ± 0.47	76.71 ± 2.33	73.55 ± 1.70	190.48 ± 5.17
6 (+α, 0)	65	15	14.48	29.40 ± 1.03	32.68 ± 2.92	25.05 ± 1.87	152.97 ± 9.06
7 (0, −α)	55	8	9.24	16.02 ± 0.27	75.81 ± 1.37	44.63 ± 0.61	187.73 ± 0.86
8 (0, +α)	55	22	20.49	19.52 ± 0.57	31.62 ± 1.45	30.21 ± 0.48	152.36 ± 1.72
9 (0, 0)	55	15	13.73	23.31± 1.98	59.90 ± 1.22	34.75 ± 1.02	184.38 ± 5.17
10 (0, 0)	55	15	14.48	21.64 ±1.58	58.88 ± 1.08	34.42 ± 1.45	152.97 ± 9.06
11 (0, 0)	55	15	12.66	26.08±2.03	60.56 ± 1.36	34.12 ± 1.25	140.78 ± 4.31
Adj. *R*^2^			0.875	0.767	0.990	0.998	0.366
*p*-value			0.0002	0.0030	<0.0001	<0.0001	0.3341

**Table 2:** Diameter inhibition zones (mm) of ME in the agar disc diffusion method.

Test Set	SFE Conditions		Inhibitory Activity of ME (mm)			
	Temperature (C°)	Pressure (MPa)	*S. aureus*	*B. subtilis*	*P. aeruginosa*	*E. coli*
1 (−1, −1)	45	10	14.56 ± 0.36	-	-	9.00 ± 0.05
2 (+1, −1)	60	10	12.11 ± 0.18	-	9.33 ± 0.71	10.33 ± 0.09
3 (−1, +1)	45	20	21.67 ± 0.27	-	7.00 ± 0.00	8.11 ± 0.03
4 (+1, +1)	60	20	17.00 ± 0.25	7.56 ± 1.51	8.11 ± 0.33	8.11 ± 0.03
5 (−α, 0)	40	15	12.22 ± 0.04	8.67 ± 1.12	-	8.00 ± 0.00
6 (+α, 0)	65	15	11.33 ± 0.05	8.33 ± 0.50	8.00 ± 0.00	8.00 ± 0.00
7 (0, −α)	55	8	15.44 ± 0.19	-	7.89 ± 1.54	14.11 ± 0.11
8 (0, +α)	55	22	11.89 ± 0.03	-	-	8.11 ± 0.06
9 (0, 0)	55	15	17.00 ± 0.13	-	7.44 ± 0.53	8.00 ± 0.05
10 (0, 0)	55	15	12.11 ± 0.08	-	8.33 ± 0.50	8.33 ± 0.07
11 (0, 0)	55	15	12.56 ± 0.09	-	8.00 ± 1.22	8.00 ± 0.05
Tetracycline (30 µg/mL)			25.89 ± 1.62	47.60 ± 0.26	12.22 ± 0.04	8.67 ± 1.12

- zone of not indicating the effectiveness of antibiotics.

**Table 3:** Minimum inhibitory concentrations (MIC) and minimum bactericidal concentrations (MBC) (%) of ME.

Treatment	SFE Conditions		MIC (%*v*/*v*)				MBC (%*v*/*v*)			
	Temperature (C°)	Pressure (MPa)	*S. aureus*	*B. subtilis*	*P. aeruginosa*	*E. coli*	*S. aureus*	*B. subtilis*	*P. aeruginosa*	*E. coli*
1 (−1, −1)	45	10	20	80	40	10	20	80	40	10
2 (+1, −1)	60	10	20	80	20	10	10	80	40	10
3 (−1, +1)	45	20	10	80	40	10	20	80	20	10
4 (+1, +1)	60	20	10	40	40	10	5	80	40	5
5 (−α, 0)	40	15	20	80	40	10	20	80	40	10
6 (+α, 0)	65	15	10	40	40	10	10	80	40	10
7 (0, −α)	55	8	10	40	40	10	10	80	40	10
8 (0, +α)	55	22	10	40	20	10	10	80	20	20
9 (0, 0)	55	15	10	40	20	10	10	80	10	5
10 (0, 0)	55	15	10	40	20	10	10	80	20	20
11 (0, 0)	55	15	10	40	20	10	10	80	20	10

**Table 4:** Essential oils chemical compositions from supercritical fluid extraction and the hydro-distillation of ME.

Compound	SFE		Hydro-Distillation	
	RT	Peak Area%	RT	Peak Area%
alpha-pinene	5.31	7.43	5.55	9.37
Sabinene	5.42	1.13	5.87	3.87
beta-pinene	6.41	23.10	6.37	11.20
Beta-myrcene	6.63	4.08	6.54	3.32
Alpha-phellandrene	7.13	6.81	7.24	8.61
d-limonene	7.73	16.08	7.84	22.94
Beta-ocimene	7.86	6.40	8.05	1.44
Gamma-terpinene	8.06	3.87	8.48	2.44
1-octanol	8.42	3.57	8.60	3.50
Cyclohexanol	9.09	1.05	9.12	0.46
Linalool	9.39	1.15	9.44	1.60
Cyclohexanal	10.22	0.55	10.23	-
Gamma-terpinene	11.08	0.50	11.10	0.66
Terpinene-4-ol	11.87	8.13	11.88	4.48
Alpha-terpinol	12.18	1.41	12.25	1.24
Octyl acetate	-	-	12.42	0.73
2-dodecanal	12.39	1.71	12.44	1.94
2-cyclohexen-1-ol	12.60	0.59	12.53	0.63
Thymol	13.50	0.28	13.53	0.99
2-undecanone	14.68	0.29	14.70	0.24
2,6-octadien-1-ol	16.95	2.38	16.98	3.18
Gemacrene	18.29	0.45	18.29	0.53
9-octadecenamide	-	-	19.84	0.31

RT (Retention time, min); peak area obtained by GC-MS of the present study.

**Table 5:** Comparison of physical and chemical properties of supercritical fluid extracted and the hydro-distilled ME.

Physical and Chemical	SFE	Hydro-Distillation
% Yield	19.19 ± 1.78 ^a^	11.80 ± 0.69 ^b^
Total phenols (mg GAE/mL)	31.54 ± 1.72 ^b^	41.19 ± 0.81 ^a^
IC_50_ DPPH (mg/mL)	26.06 ± 0.00 ^b^	77.38 ± 0.01 ^a^
IC_50_ ABTS (mg/mL)	19.90 ± 0.00 ^b^	31.29 ± 0.00 ^a^

Different letters indicate the significant difference between the extraction methods (*p* < 0.05).

**Table 6:** Comparison of antimicrobial properties of supercritical fluid extracted and hydro-distilled ME.

Bacteria	Inhibition Zone (mm)		MIC (%*v*/*v*)		MBC (%*v*/*v*)	
	SFE	Hydro- Distillation	SFE	Hydro- Distillation	SFE	Hydro- Distillation
*B. subtilis*	8.17 ± 0.41 ^b^	12.11 ± 0.93 ^a^	40	40	80	40
*S. aureus*	11.50 ± 0.84 ^b^	28.90 ± 0.18 ^a^	5	0.625	10	0.625
*P. aeruginosa*	8.67 ± 0.52 ^b^	20.11 ± 0.33 ^a^	10	10	20	5
*E. coli*	9.00 ± 0.63 ^b^	27.00 ± 0.13 ^a^	2.5	2.5	5	1.25

Different letters indicate the significant difference between the extraction methods for the inhibition zone (*p* < 0.05).

## Data Availability

Data will be supplied as requested.

## Supplementary Materials

The following supporting information can be downloaded at: https://www.mdpi.com/article/10.3390/plants12112211/s1, Figure S1. Contour plots of ME in terms of a) Yield (%) (b) Total phenolic compounds (c) DPPH, (d) ABTS, and (e) FRAP as a function of temperature and pressure. Figure S2. Design of experiment for the extraction of Makwaen pepper using SFE and hydro-distillation extraction.: title; Table S1: Regression equations, coefficient of determinations (*R*^2^), and *p* values of each response of ME using SFE. Table S2. Pearson Correlation for total phenolic compounds with antioxidation.
