# Supplementary material for: Antioxidant and Antimicrobial Properties and GC-MS Chemical Compositions of Makwaen Pepper (Zanthoxylum myriacanthum) Extracted Using Supercritical Carbon Dioxide
Source: Plants (Basel). 2023 Jun 3;12(11):2211. doi: 10.3390/plants12112211 (PMC10255492; doi:10.3390/plants12112211)
Supplement: Supplementary file 1 [file plants-12-02211-s001.zip › plants-2395092_Supplement_2023.06.02.pdf]

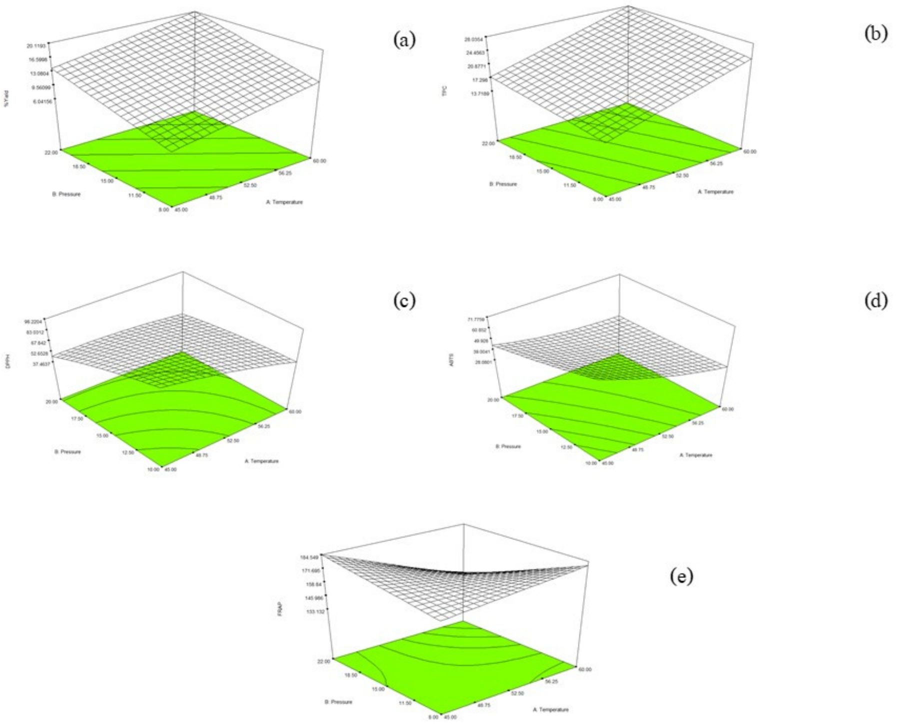

Figure S1. Contour plots of ME in terms of a) Yield (%) (b) Total phenolic compounds (c) DPPH, (d) ABTS, and (e) FRAP as a function of temperature and pressure.

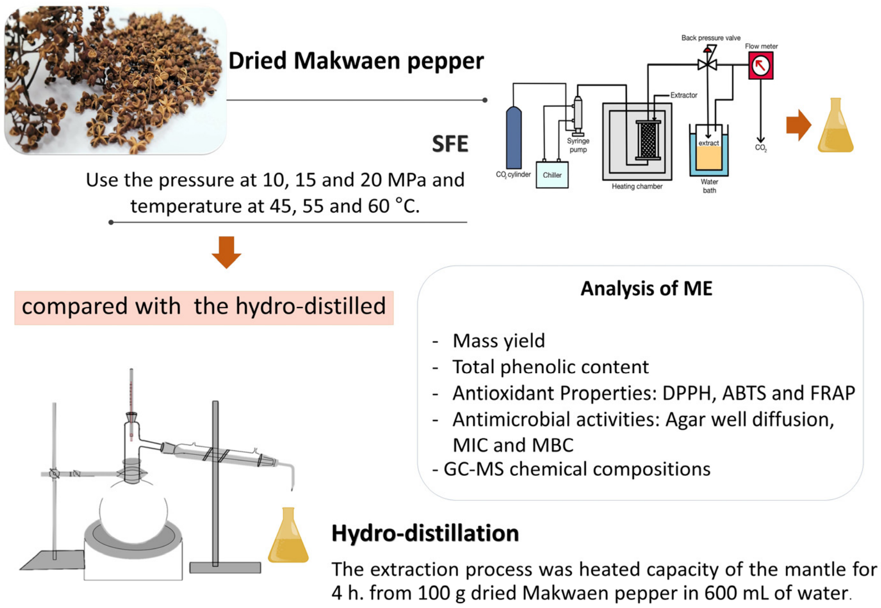

Figure S2. Design of experiment for the extraction of Makwaen pepper using SFE and hydro-distillation extraction.

Table S1. Regression equations, coefficient of determinations (R^2), and p values of each response of ME using SFE.

Responses	Regression equation	Adj R^2	p-value
Yield (%)	$-17.15 + 0.41 * X_1 + 0.55 * X_2$	0.875	0.0002
Total phenols (mg GAE/mL)	$-19.83 + 0.70 * X_1 + 0.28 * X_2$	0.767	0.0030
IC_{50} DPPH (µg/mL extract)	$210.02 + 0.97 * X_1 - 13.08 * X_2 - 0.06 * X_1^2 - 0.11 * X_2^2 + 0.25 * X_1 * X_2$	0.990	<0.0001
IC_{50} ABTS (µg/mL extract)	$491.90 - 11.46 * X_1 - 12.26 * X_2 + 0.07 * X_1^2 + 0.05 * X_2^2 + 0.18 * X_1 * X_2$	0.998	<0.0001

p-value indicates a significant difference at 95% confidence ($p < 0.05$). **X_1= Temperature, X_2= Pressure.

Table S2. Pearson Correlation for total phenolic compounds with antioxidation.

	Correlations (Pearson Correlation)			
	**TPC**	**DPPH**	**ABTS**	**FRAP**
**TPC**	1			
**DPPH**	-0.654*	1		
**ABTS**	-0.783*	0.841*	1	
**FRAP**	-0.279	0.250	0.302	1

* indicates a significant difference at 95% confidence ($p < 0.05$).
